# Analysis of the Enantioselective Effects of PCB95 in Zebrafish (*Danio rerio*) Embryos through Targeted Metabolomics by UPLC-MS/MS

**DOI:** 10.1371/journal.pone.0160584

**Published:** 2016-08-08

**Authors:** Nana Xu, Pengqian Mu, Zhiqiang Yin, Qi Jia, Shuming Yang, Yongzhong Qian, Jing Qiu

**Affiliations:** Institute of Quality Standard & Testing Technology for Agro-Products, Key Laboratory of Agro-product Quality and Safety, Chinese Academy of Agricultural Sciences, Ministry of Agriculture, Beijing 100081, China; University of Iowa, UNITED STATES

## Abstract

As persistent organic pollutants, polychlorinated biphenyls (PCBs) accumulate in the bodies of animals and humans, resulting in toxic effects on the reproductive, immune, nervous, and endocrine systems. The biological and toxicological characteristics of enantiomers of chiral PCBs may differ, but these enantioselective effects of PCBs have not been fully characterized. In this study, we performed metabolomics analysis, using ultra-high performance liquid chromatography tandem mass spectrometry (UPLC-MS/MS) to investigate the enantioselective toxic effects of PCB95 in zebrafish (*Danio rerio*) embryos after exposure to three dose levels of 0.1, 1, and 10 μg/L for 72 h. Multivariate analysis directly reflected the metabolic perturbations caused by PCB95. The effects of (-)-PCB95 and (+)-PCB95 were more prominent than those of the racemate in zebrafish embryos. A total of 26 endogenous metabolites were selected as potential marker metabolites with variable importance at projection values larger than 1 and significant differences (*p*<0.05). These metabolites included amino acids, organic acids, nucleosides, betaine, and choline. The changes in these biomarkers were dependent on the enantiomer-specific structures of PCB95. Fifteen metabolic pathways were significantly affected, and several nervous and immune system-related metabolites were significantly validated after exposure. These metabolic changes indicated that the toxic effects of PCB95 may be associated with the interaction of PCB95 with the nervous and immune systems, thus resulting in disruption of energy metabolism and liver function.

## Introduction

Polychlorinated biphenyls (PCBs) are ubiquitous contaminants in the environment and can accumulate in the bodies of animals and humans, resulting in toxicity to the reproductive, immune, nervous and endocrine systems [1]. For example, in Japan 1,684 people were poisoned after eating rice bran oil contaminated with PCBs, and more than 30 people died in 1968 [2]. Although PCBs became a main priority of the Stockholm International Convention in 2001 as a persistent organic pollutant (POPs) [3], PCBs present continued risks because of their persistence, refractory nature, and ease of accumulation.

Of the 209 PCBs congeners, 78 display axial chirality in their nonplanar conformation, and 19 are stable at ambient temperatures. Enantiomers of chiral PCBs have different biological and toxicological characteristics. Interestingly, the enantiomer fraction (EF) of PCB95 has opposite effects in fish (EF<0.5) and bivalves (EF >0.5) [4]. Moreover, enantiomers of PCB139 exhibit different potencies in inducing cytochrome P450 activity [5]. Therefore, it is necessary to evaluate the enantioselective effects of chiral PCBs on environmental problems and their risk to human health and the ecological environment.

Metabolomics is a relatively new omics technology involving the comprehensive measurement of small-molecule metabolites in organisms and has been widely used in the study of drug toxicity mechanisms and disease. Rat and zebrafish have recently been used as models for metabolomic and genomic research [6,7], similarly to research on human diseases such as cancer [8] and neurodegenerative diseases [9]. Several metabolomic studies of the effects of PCBs in rats have demonstrated that PCBs and tetrachlorodibenzo—p-dioxin (TCDD) can lead to immune system disturbances, liver and nervous system dysfunction, and lipid metabolism perturbation [10]. However, few studies have reported metabolomics analysis of PCB enantiomers in organisms.

Therefore, in this study, we used ultra-high performance liquid chromatography and tandem mass spectrometry (UPLC-MS/MS) to study the enantioselective toxic effects of chiral PCB95 in zebrafish (*Danio rerio*) embryos based on metabolomics.

## Materials and Methods

### Reagents and materials

A Milli-Q water purification system (Millipore, France) was used to prepare deionized water (18.2 MΩ). HPLC-grade 96% formic acid (MREDA, USA) and acetonitrile (Fisher Scientific, USA) were used as solvents. Methanol, *n*-hexane, and dichloromethane were HPLC grade and purchased from Fisher Scientific (USA). A mixed standard solution consisting of 26 compounds, including amino acids, organic acids, amines, and choline, was purchased from the National Institute of Metrology (China). The PCB95 standard was purchased from AccuStandard (purity>98%; USA).

The enantiomers of chiral PCB95 were separated and quantified by Waters 2695 HPLC with an ultraviolet detector (USA) at 220 nm and a Lux 5 μm cellulose-3 column (250mm×4.6mm×5 μm; Phenomenex, USA) at 30°C. The flow rate was 1 mL/min, and the mobile phase was 100% of *n*-hexane. The injection volume was 20 μL. The order of elution of the enantiomers of PCB95 was confirmed using an online optical rotation detector (IBZMESSTECHNIK, Germany). The first eluted enantiomer was (-)-PCB95, and the second enantiomer was (+)-PCB95. The purity of the two enantiomers was greater than 98%. This method was validated previously in a comprehensive study [11].

### Sample collection and preparation

#### Fish husbandry and embryo collection

Specific pathogen-free AB line zebrafish (*Danio rerio*) were cultured in a temperature-controlled laboratory (28±0.5°C) with a 14h:10h light/dark cycle. The conductivity of the water used for the zebrafish was 480+20 μS/cm, and the pH was adjusted to between pH 6.5 and 8.5 [12]. The fish were fed fresh frozen Artemia (Binhaijun Industry Co. Ltd, China) three times daily. Embryos were collected within 0.5 h post fertilization (hpf; male/female ratio of 1:1 or 1:2) and were rinsed with water.

#### Exposure of zebrafish embryos

This study was performed in conformity with Chinese legislation and approved by the independent animal ethics committee at the Chinese Academy of Agricultural Sciences.

Chemical exposure was initiated at 5–6 hpf. The embryos were randomly allocated to three exposure groups: the racemic group, (-)-PCB95 group, and (+)-PCB95 group. The three exposure groups were each treated with three dose levels (high, middle, and low), yielding a total of 10 treatment groups, including one control group. All of the treatment groups were performed in ten replications (10 plastic petri dishes per group), with 20 embryos per sample and 200 embryos per treatment group. Zebrafish embryos were exposed for 72 h at 28 ± 0.5°C with a 14 h: 10 h light/dark cycle in constant temperature incubator. The treatment volume is 40 mL per petri dish, and the exposure solutions were completely renewed every 24 h [13]. Acetone was used as the solvent to prepare the stock solution, and the solvent concentration was 0.01%. The high-, middle-, and low-dose test solutions were 10, 1 and 0.1 μg/L respectively, and the control was only treated with 0.01% acetone. After the 72 h treatment, the embryos were collected in 1.5 mL tubes, washed three times, and snap-frozen in liquid nitrogen, and then stored at -80°C until extraction.

#### Sample preparation

The embryo samples were thawed at room temperature and thoroughly homogenized in 100 μL of cold methanol on ice. Next, 140 μL of cold methanol, 195 μL of distilled water, and 240 μL of dichloromethane were added to the homogenate. The samples were then shook for 1 min, incubated on ice for 10 min, and centrifuged at 12000× *g* for 5 min at 4°C to remove protein and tissue debris [14,15]. After centrifugation, 100 μL of the upper polar layer was transferred to a 1.5-mL glass vial and diluted 10-fold with methanol/water (50:50, v/v) containing 0.25% formic acid for UPLC-MS/MS analysis.

To evaluate the reliability of the preparation method, a standard solution containing 26 different types of compounds was added to blank extraction solvent at concentrations of 20, 100 or 200 μg/L before preparation. A quality control (QC) sample was prepared by mixing equal volumes from each embryo sample after preparation. The QC sample and the standard solution were analyzed before and during the sequence to estimate the method reliability.

### UPLC-MS/MS analysis

UPLC-MS/MS analysis was performed using a Waters UPLC system with a QTrap 6500 mass spectrometer (AB SCIEX, USA) in positive ion mode. The secondary mass spectrum information for 76 small polar compounds selected from the MassBank (www.massbank.jp/) database based on references was used for the MRM scanning module. The prepared samples were injected into an Xbridge C18 column (4.6mm×150mm×3.5 μm, Waters) at 25°C. The flow rate was 300 μL/min, and the mobile phase consisted of water with 0.1% formic acid (A) and acetonitrile (B). The gradient conditions were 5% B at 0–2 min, 5%–60% B over 2–10 min, and 60%–5% B over 10–16 min.

### PCB95 analysis in water

To ensure the actual concentrations were consistent with the nominal concentrations, the water samples were measured at 0 and 24 h with 3 replications. Water (100 mL) was extracted with 20 ml of *n*-hexane in a separating funnel. The up-layer was transferred into 50-mL polyethylene centrifuge tube and dried by nitrogen at 35°C. Finally, the residue was redissolved in 0.1 mL of *n*-hexane for gas chromatography equipped with electron capture detector (GC-ECD) analysis. The GC-ECD (Agilent 7890 A; USA) analysis was performed using chiral Chirasil-Dex column (25 m×0.25 mm×0.25 μm). The injection temperature was 260°C with splitless stream sampling, and temperature program was as follows: 60°C hold for 2 min, 10°C/min to 150°C, 1°C/min to 180°C and hold for 20 min.

### Data analysis

MarkerView1.2.1 (AB SCIEX) was used to extract peak information, regulation of migration time, and normalization. The normalized data were imported into SIMCA-P 11.0 software (Umetrics, Sweden) for multivariate analysis and IBM SPSS21.0 software (Chicago, IL, USA) for univariate analysis. The MetaboAnalyst 3.0 web service was used to perform pathway enrichment analysis and pathway topology analysis.

## Results and Discussion

### Method validation

**Fig 1:**
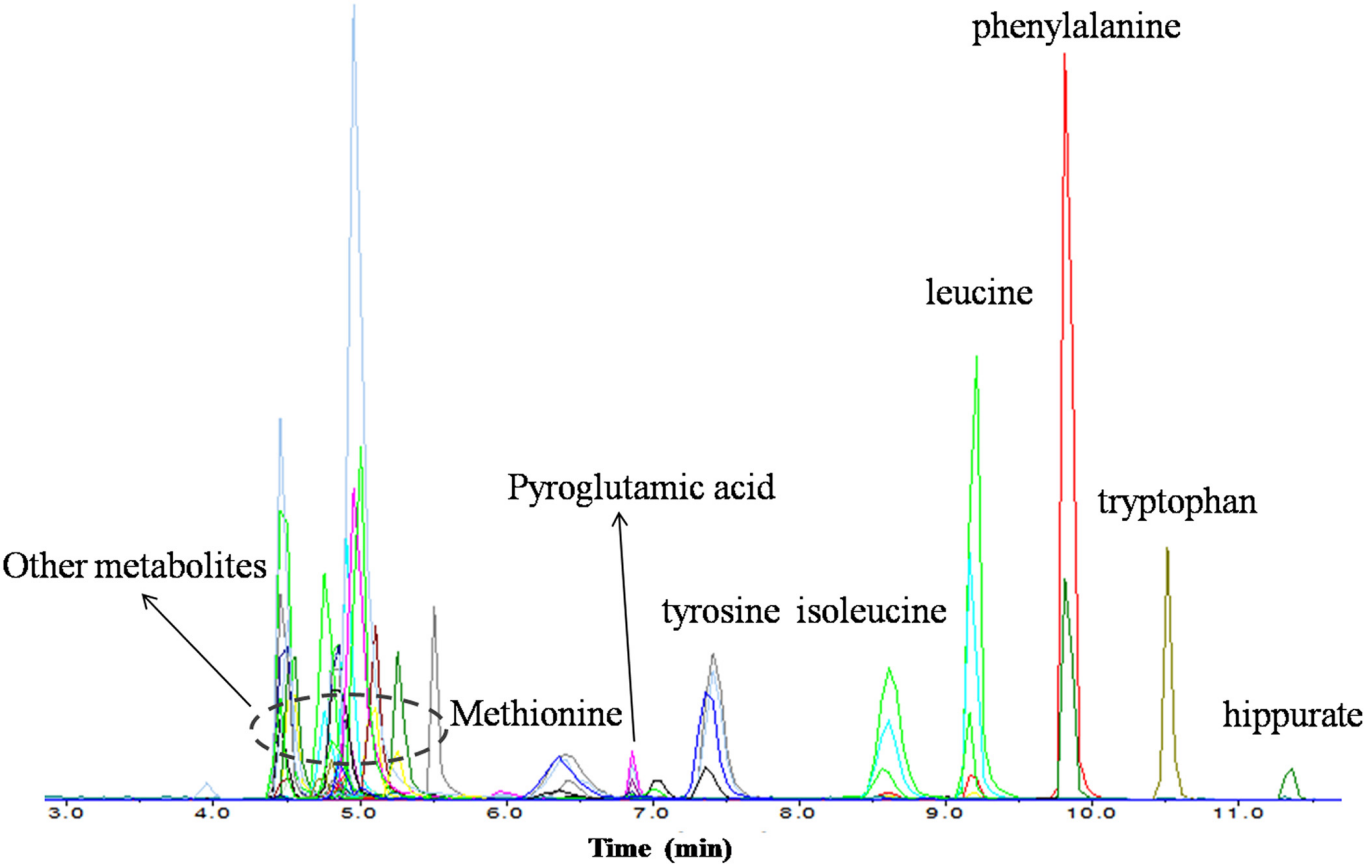
Typical UPLC-MS/MS chromatogram of 60 polar metabolites in zebrafish embryos exposed to low-dose (-)-PCB 95.

To evaluate whether this method could be useful for the detection of diverse endogenous metabolites, a standard solution composed of 26 different standards, including amino acids, organic acids, amines, and choline, was analyzed under defined UPLC-MS/MS conditions before analyzing sequences of real samples. All compounds in the standard mixtures with the exception of glycine were clearly separated, suggesting that the UPLC-MS/MS method was suitable for analyzing a variety of metabolites. Of the 76 metabolites selected from the MassBank (www.massbank.jp/) database, 60 metabolites were detected in the embryos. As shown in Fig 1, most of the metabolites were eluted at 4 to 6 min; methionine, tyrosine, and pyroglutamic acid were eluted at 6 to 8 min. As isomers, leucine, and isoleucine were eluted at about 9 min. Tryptophan, phenylalanine, and hippurate have a common characteristic, containing a benzene ring, and they were eluted after 10 min. All of the metabolites were eluted before 12 min, and this rapid method fulfilled the requirements for adequate analysis of polar metabolites.

The reliability of the preparation method was evaluated by measuring the recoveries of 26 standard compounds. Their recoveries ranged from 71.8% to 116%, except for glycine. Therefore, the preparation method was considered effective. The stability of analysis method was evaluated by relative standard deviation (RSD) of QC samples (*n* = 13). The RSDs were less than 30% for 53 metabolites, and the variation ranges in all QC samples were within 2 times the standard deviation (SD). The results showed that the above analytical methods were reliable and stable to explore organism information.

In water samples, a satisfactory recovery was obtained from 93.2–108.5% with RSDs of 4.3–12.8% at three spiked concentration levels (0.1, 1, and 10 μg/L). As shown in Table 1, the actual concentrations of PCB95 were no less than 80% of the nominal concentrations within 24 hours and biotransformation was not found for the two enantiomers in water. So the concentrations of PCB95 were stable and there was no obvious difference in uptake during abiotic processes.

**Table 1:** The concentrations of PCB95 in water samples.

Test Time	Theoretical concentration (μg/L)	Actual concentration (μg/L)		
		Racemic exposure	(-)-PCB95 exposure	(+)-PCB95 exposure
0 h	0.1	0.09	0.11	0.11
	1	1.03	0.98	1.15
	1 0	10.12	9.84	10.30
24 h	0.1	0.08	0.09	0.09
	1	0.91	0.93	0.87
	1 0	8.91	9.40	9.05

### Multivariate and univariate data analysis

To directly analyze changes in the metabolome following treatment with PCB95, a supervised multivariate data analysis method, i.e., partial least squares discriminant analysis (PLS-DA), with Par-scaling was applied to the preprocessed data. This method was used to predict correlations between individual and control samples. PLS-DA is based on the PLS model, in which the dependent variable is selected to represent a class. PLS-DA is a powerful method for discrimination analysis between different groups and for identifying marker components that can explain differences among samples [16,17].

**Fig 2:**
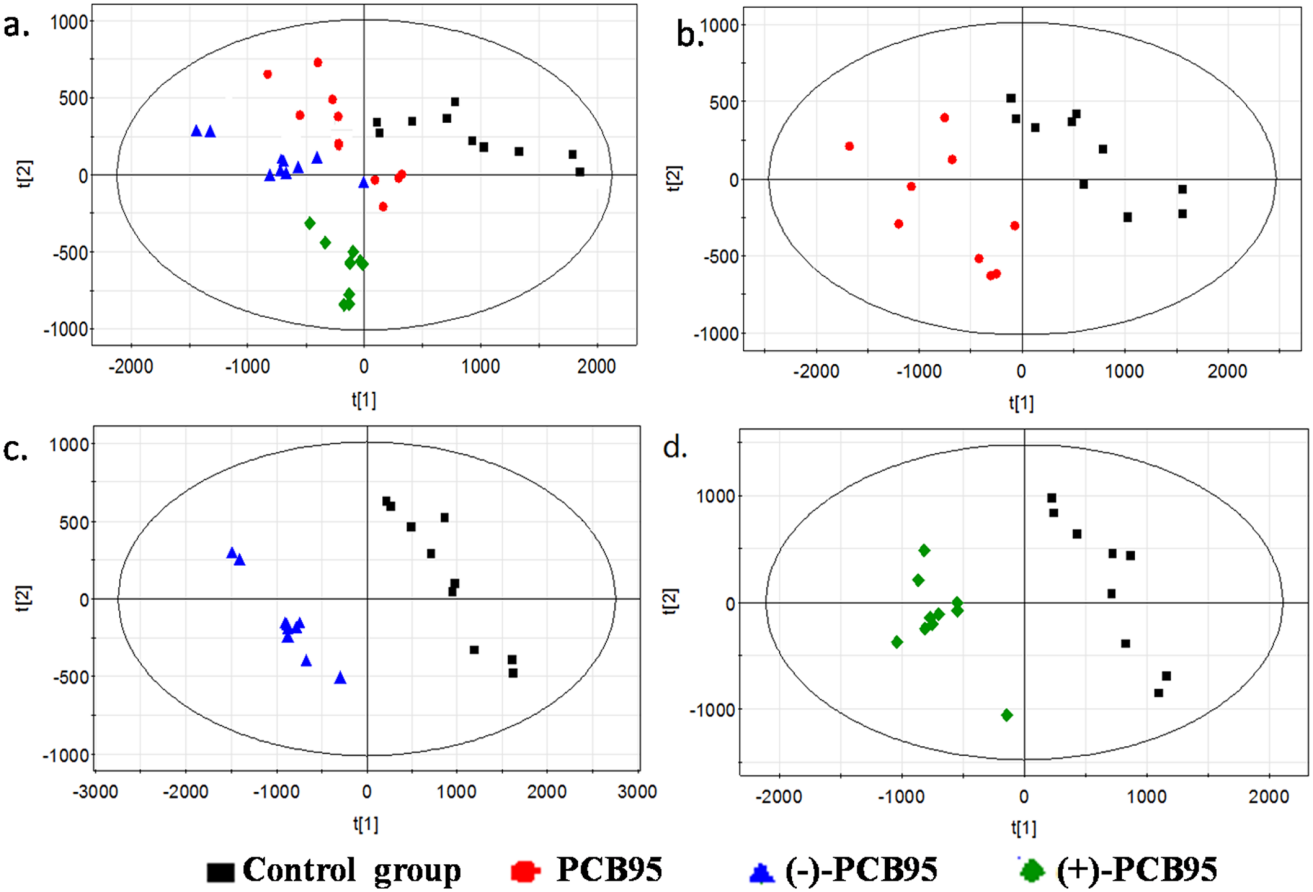
Partial least squares discriminant analysis (PLS-DA) score plots of data at 1 μg/L for all four groups (a), the control group versus the racemic group (b), the control group versus the (-)-PCB95 group (c), and the control group versus the (+)-PCB95 group (d).

The PLS-DA results (Fig 2) indicated that all PCB95-treated groups clustered at the same side and were clearly separated from the control group with R^2^X>0.724 and Q^2^>0.691. Groups exposed to PCB95 exhibited a clear separation trend, indicating that the differences between (+)- or (-)-PCB95 and the control group were more remarkable than the difference between the racemic group and the control group. The metabolic perturbations induced by different forms of PCB95 also occurred in the low-dose group (0.1 μg/L). It can be inferred that the effects of the two enantiomers of PCB95 may have been weakened when administered together, and further studies are needed to examine these variations.

To identify marker metabolites for these variations, variable importance in projection (VIP) analysis was performed to assess the importance of variables in the model with regard to Y [18]. If the VIP value was greater than 1, the variable was selected for further study. A total of 26 metabolites that differed between the treated and control groups were selected, and the VIP results were confirmed by S-plot analysis. In an S-plot, ideal biomarkers have high magnitude and high reliability, indicating a smaller risk of spurious correlations [19]. All of the selected metabolites were distributed on the edge of the S-plots with high magnitude and high reliability, indicating that these two types of analysis were in agreement with each other. Statistical analysis of variance (ANOVA) was then performed to select metabolites that differed significantly between groups (*p*<0.05). Finally, we identified and quantified 22 metabolites from 26 potential markers using standards. The details of the potential markers are shown in Table 2.

**Table 2:** Detailed information of biomarkers in the PCB95-treated groups compared with the control group.

Common Name	MRM ion pair (m/z)		Retention Time	Chemical Formula
Aspartate acid^a^	134.045/116.035	134.045/88.040	4.86	C_4_H_7_NO_4_
Glutamic acid^a^	148.200/102.000	148.200/84.100	4.92	C_5_H_9_NO_4_
Serine^a^	106.100/60.200		4.80	C_3_H_7_NO_3_
Histidine^a^	156.007/110.070		4.56	C_6_H_9_N_3_O_2_
Threonine^a^	120.100/74.100		4.88	C_4_H_9_NO_3_
Arginine^a^	175.200/70.000		4.57	C_6_H_14_N_4_O_2_
Alanine ^a^	90.056/44.049		4.88	C_3_H_7_NO_2_
Tyrosine^a^	182.080/165.050	182.080/136.076	7.67	C_9_H_11_NO_3_
Valine^a^	118.100/72.200		5.85	C_5_H_11_NO_2_
Methionine^a^	150.200/132.951	150.200/104.100	6.86	C_5_H_11_NO_2_S
Phenylalanine^a^	166.090/103.056	166.090/120.080	9.86	C_9_H_11_NO_2_
Leucine^a^	132.100/86.100		9.18	C_6_H_13_NO_2_
Isoleucine^a^	132.100/86.200	132.100/69.070	8.60	C_6_H_13_NO_2_
Lysine^a^	147.100/84.100	147.100/130.000	4.53	C_6_H_14_N_2_O_2_
Proline^a^	116.070/70.000		5.11	C_5_H_9_NO_2_
Cysteine^a^	241.031/151.983	241.031/120.011	4.73	C_6_H_12_N_2_O_4_S_2_
Tryptophan^a^	205.200/188.300		10.60	C_11_H_12_N_2_O_2_
Taurine^a^	126.022/108.010	126.022/90.700	4.88	C_2_H_7_NO_3_S
Glutamine^a^	147.077/130.050	147.077/84.045	4.82	C_5_H_10_N_2_O_3_
γ-Aminobutyrate^a^	104.070/87.040	104.070/58.066	4.88	C_4_H_9_NO_2_
Choline^a^	104.000/60.000		4.80	C_5_H_14_NO
Pyroglutamic acid^a^	130.050/112.900	130.050/84.045	7.10	C_5_H_7_NO_3_
IMP	349.055/137.046	349.055/119.036	5.36	C_10_H_13_N_4_O_8_P
Pipecolic acid	130.100/84.100	130.100/56.200	4.46	C_6_H_11_NO_2_
Betaine	118.087/58.100	118.087/59.073	4.99	C_5_H_11_NO_2_
N-Acetylornithine	175.100/115.300	175.100/70.000	4.52	C_7_H_14_N_2_O_3_

^a^ Represents the metabolites identified by standards.

### Dose-response relationship and enantioselective toxic effects of PCB95

**Fig 3:**
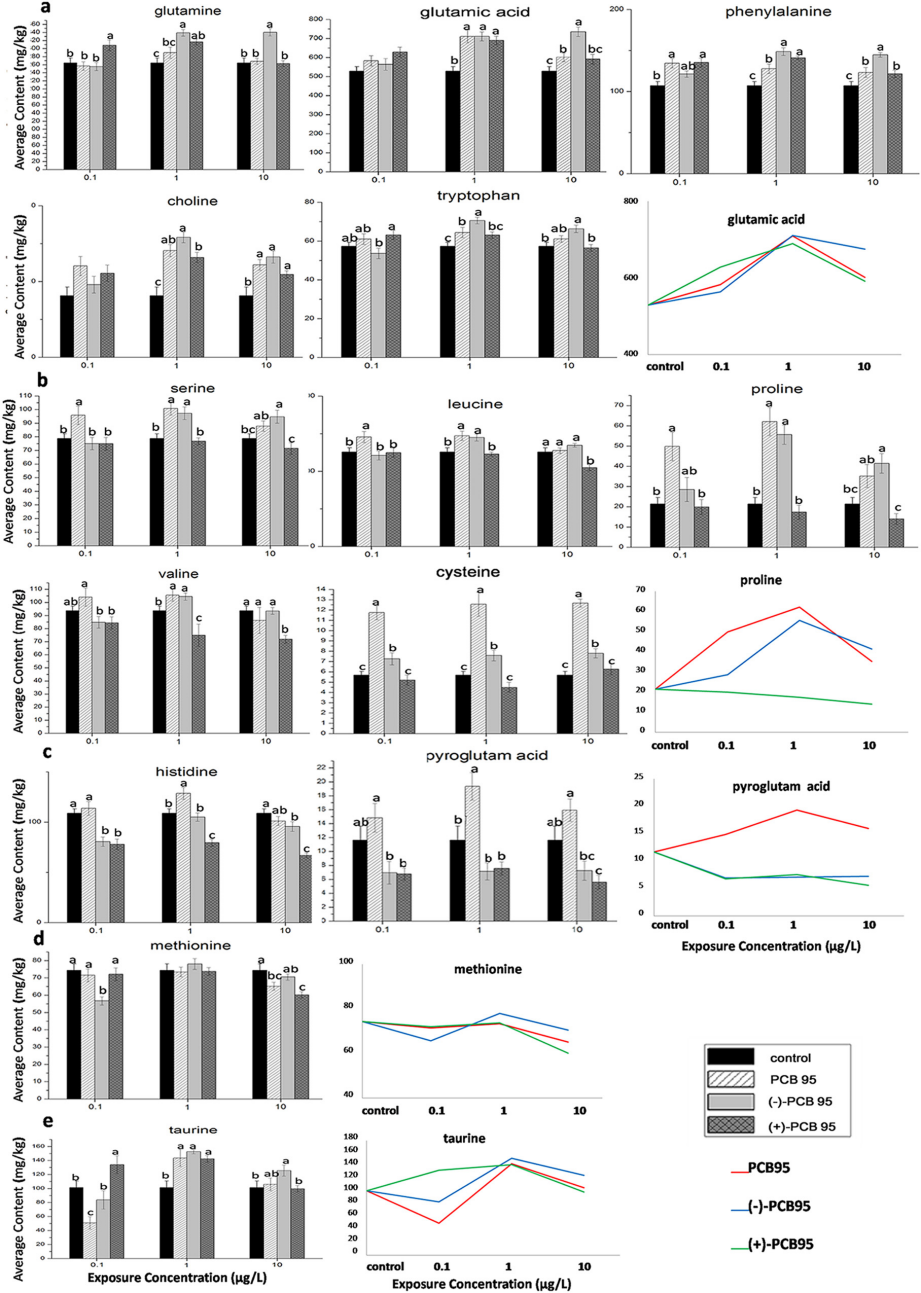
Average concentrations of typical significant metabolites with bigger variation trends in zebrafish embryos treated with different forms of chiral PCB95. The metabolites were separated into five groups (a, b, c, d and e) based on similar dose-response patterns, and different letters on the top of columns means significant differences with P<0.05 (ANOVA).

Significant metabolites were divided into 5 groups according to similar dose-response patterns, and each group has a common variation trend (Fig 3). To compare the relative contents of significant compounds in different treatment groups, average concentrations were graphed as histograms. To more intuitively descript the variation trend of each group, line chart of the representative metabolite was presented on the last panel. The most typical metabolites in each group were showed in Fig 3 for easier understand. For Group a, including aspartate acid, phenylalanine, tryptophan, glutamine, glutamic acid, tyrosine, γ-aminobutyrate, betaine, choline, IMP, and N-acetylornithine, their average concentrations were higher in the PCB95-treated groups than those in the control group. These metabolites increased gradually from the control group to the middle-dose group, and then slightly decreased to the high-dose group (Fig 3a). These data demonstrate that low and middle doses induced the stress response in zebrafish embryos, and damage may occur to the embryos at high doses. For Group b, including serine, leucine, isoleucine, lysine, proline, valine, alanine, cysteine, threonine, and pipecolic acid, their average concentrations increased gradually from the control to the middle-dose group, and decreased to the high-dose group in the racemic and (-)-PCB95 groups. In contrast, these compounds were at equal or lower levels in the (+)-PCB95 group than those in the control group (Fig 3b). Therefore, these compounds may be labeled as markers for (+)-PCB95. However, further studies are needed to confirm this hypothesis. The levels of pyroglutamic acid and histidine were increased in the racemic group compared with the control group but decreased after only exposure to (-)-PCB95 or (+)-PCB95 (Fig 3c). As the dose of PCB95 gradually increased, methionine levels decreased only in high dose groups (Fig 3d). However, taurine exhibited complex variation patterns in different groups (Fig 3e).

The variation tendencies of potential markers among different groups at a concentration of 1 μg/L are shown in Table 3. Alanine and threonine significantly increased in the racemic and (-)-PCB95 groups compared with the control group. In contrast, they decreased in the (+)-PCB95 group compared with other groups. These results not only indicate that (-)-and (+)-PCB95 have opposite effects on alanine and threonine but also predict that (-)-PCB95 plays a more important role when the two enantiomers are administered simultaneously. Glutamic acid, taurine, γ-aminobutyrate, choline, betaine, and phenylalanine increased when embryos were exposed to PCB95, and some of these metabolites increased more in the (-)-PCB95 group than in the other two groups, revealing that the three forms of PCB95 have a similar effect, but the effect of (-)-PCB95 is more intense. Tryptophan, valine, serine, leucine, isoleucine, lysine, proline, and pipecolic acid increased in the racemic and (-)-PCB95 groups but did not differ significantly between the control and (+)-PCB95 groups. Pyroglutamic acid, cysteine, and histidine significantly increased only in the racemic group, which suggests that although a single enantiomer had little influence, the effect is enhanced when the two enantiomers were present as the racemate. In the (+)- and (-)-PCB95 groups, glutamine, tyrosine, and IMP were simultaneously increased; the stable levels of these metabolites in the racemic group indicated antagonism between the two enantiomers. However, aspartate acid increased only in (+)-PCB95 groups compared to the control groups. The differences in above endogenous metabolites suggested enantioselective toxicities between two enantiomers of PCB95.

**Table 3:** Metabolites selected as biomarkers in the groups treated with chiral PCB95 at a concentration of 1 μg/L.

Common Name	CK vs rac-PCB95			CK vs (-)-PCB95			CK vs (+)-PCB95			rac- vs (-)-PCB95			rac- vs (+)-PCB95			(-)- vs (+)-PCB95		
	VIP	*p*	Change^b^	VIP	*p*	Change^b^	VIP	*p*	Change^b^	VIP	*p*	Change^b^	VIP	*p*	Change^b^	VIP	*p*	Change^b^
Alanine^a^	1.082	0.015	↑	1.221	0.003	↑	3.417	0.004	↓				1.202	0.000	↓	0.960	0.000	↓
Threonine^a^	1.033	0.005	↑	0.947	0.026	↑	0.976	0.043	↓				0.959	0.000	↓	0.800	0.000	↓
Glutamic acid^a^	2.116	0.000	↑	1.956	0.000	↑	0.956	0.000	↑									
Taurine^a^	2.829	0.001	↑	3.378	0.000	↑	3.352	0.001	↑									
γ-Aminobutyrate^a^	0.961	0.000	↑	0.980	0.000	↑	1.053	0.000	↑							1.003	0.001	↓
Choline^a^	1.585	0.000	↑	1.588	0.000	↑	0.966	0.000	↑							1.341	0.034	↓
Betaine	2.319	0.012	↑	2.589	0.000	↑	2.515	0.003	↑	1.871	0.022	↑				2.023	0.029	↓
Phenylalanine^a^	2.559	0.003	↑	2.868	0.000	↑	2.17	0.000	↑	2.281	0.003	↑	1.481	0.048	↑			
Tryptophan^a^	1.020	0.021	↑	1.071	0.000	↑				0.959	0.041	↑				1.001	0.015	↓
Valine^a^	1.002	0.017	↑	0.773	0.029	↑							1.808	0.000	↓	1.577	0.000	↓
Serine^a^	0.951	0.000	↑	1.008	0.002	↑							1.006	0.000	↓	0.783	0.001	↓
Leucine^a^	3.620	0.004	↑	1.479	0.009	↑							1.065	0.001	↓	3.096	0.003	↓
Isoleucine^a^	1.256	0.001	↑	1.041	0.005	↑							1.052	0.000	↓	0.911	0.000	↓
Lysine^a^	2.073	0.047	↑	2.135	0.037	↑							1.977	0.003	↓	2.281	0.002	↓
Proline^a^	2.254	0.000	↑	1.580	0.000	↑							2.648	0.000	↓	2.421	0.000	↓
Pipecolic acid	1.177	0.041	↑	1.125	0.011	↑												
Pyroglutamic acid^a^	0.916	0.005	↑							1.734	0.000	↓	1.597	0.000	↓			
Cysteine^a^	0.916	0.000	↑							1.237	0.000	↓	1.590	0.000	↓	1.879	0.004	↓
Histidine^a^	1.193	0.004	↑				2.760	0.000	↓	0.865	0.001	↓	0.877	0.000	↓	0.979	0.000	↓
Aspartate acid^a^							2.038	0.015	↑							1.002	0.016	↑
Glutamine^a^				2.164	0.000	↑	1.578	0.001	↑	1.507	0.002	↑						
Tyrosine^a^				1.539	0.000	↑	1.505	0.028	↑	1.336	0.017	↑						
IMP				0.957	0.026	↑	1.564	0.000	↑				1.507	0.000	↑	2.215	0.008	↑

^a^ Represents the metabolites identified by standards.

^b^ “↑”indicates metabolites that were increased; “↓”indicates metabolites that were decreased

In previous studies of chiral PCB95, enantioselective capacity was observed in other species. For example, the EFs of PCB95 has opposite effects in fish (EF<0.5) and bivalves (EF >0.5)[4]. Non-racemic enrichment of PCB95 was observed in striped dolphins form the Mediterranean Sea[20]. PCB95 are enantioselectively metabolized to OH-PCB95 by cytochrome P450 enzymes in Rat Liver Microsomes[21]. Therefore, differential uptake or metabolism of PCB95 enantiomers might be one of the reasons leading to differential metabolite profiles in embryo-larvae zebrafish. However, the actual reasons need to be explored by more experiments in the future.

The experimental results showed that PCB95 treatments have not obvious effects on survival and morbidity of zebrafish larvae, because the investigated cycle was relative short, the number of dead larvae was less than three and the death occurred in one or two of ten replicates of a treated group.

### Pathway analysis of the toxic effects of PCB95

**Fig 4:**
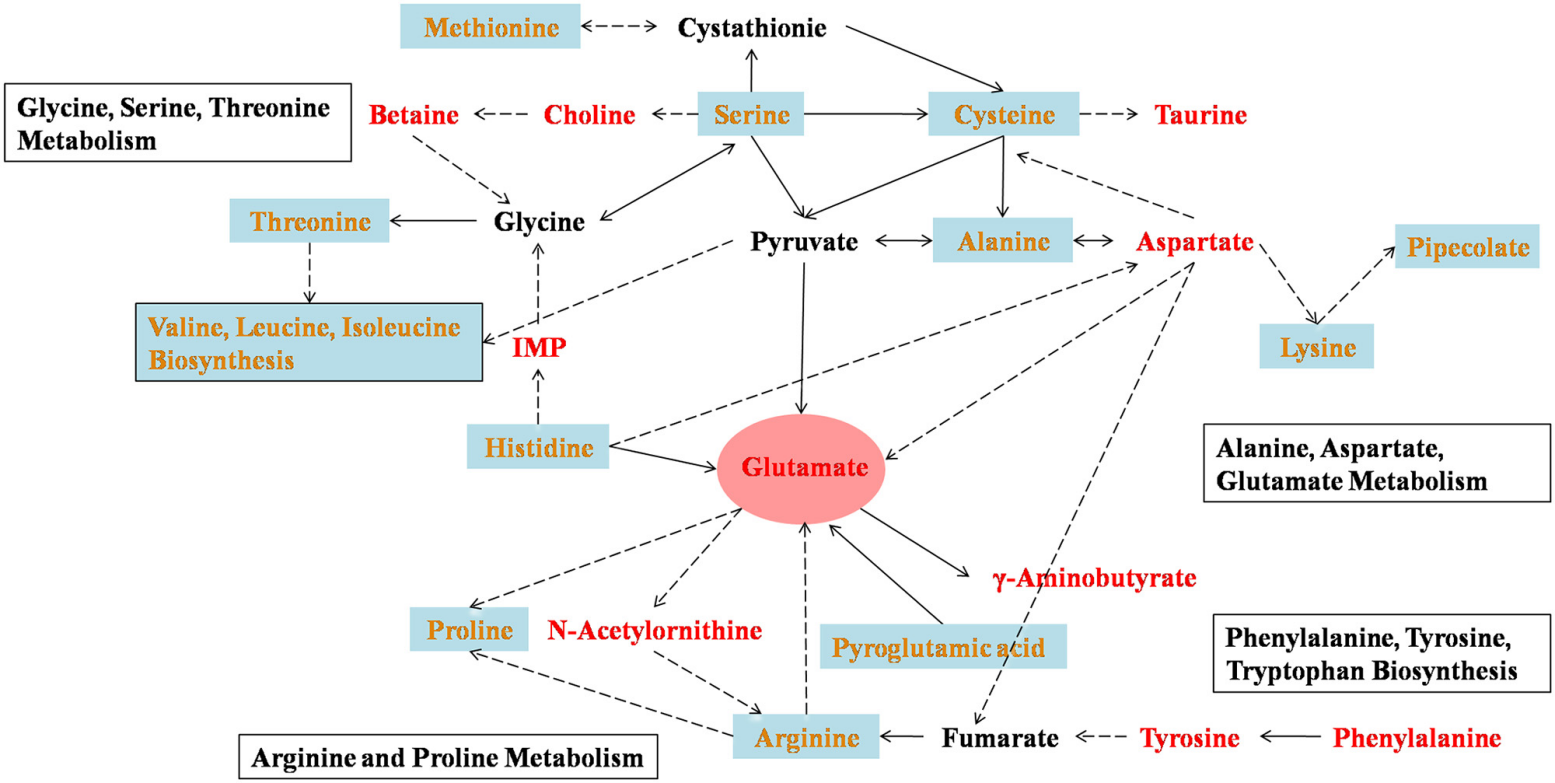
Overview of the metabolic pathway network in zebrafish embryos perturbed by PCB95. Red-labeled metabolites increased in all PCB95-treated groups, and yellow-labeled metabolites increased and decreased (or stable) across different groups because of enantioselectivity. The solid lines represent direct metabolic reactions, and the dotted lines represent multiple reactions and indirect connections between two metabolites.

Metabolic pathway analysis by MetaboAnalyst 3.0 was used to determine the most relevant pathways affected by PCB95. The results suggested that fifteen metabolic pathways were measurably perturbed, including glutathione metabolism, phenylalanine, tyrosine, and tryptophan biosynthesis, phenylalanine metabolism, and valine, leucine, and isoleucine biosynthesis (Table 4). The relevant biomarkers and disturbed pathways were summarized from KEGG (www.genome.jp/kegg/) to demonstrate the perturbation in zebrafish embryos after exposure (Fig 4).

**Table 4:** Disrupted metabolic pathways in zebrafish embryos based on KEGG analysis.

No.	KEGG Pathway	Hits^a^	p^b^	FDR	Impact^c^
1	Glutathione metabolism	1	1.56×10^−09^	4.05×10^−08^	0.01
2	Phenylalanine, tyrosine, and tryptophan biosynthesis	2	9.81×10^−07^	6.38×10^−06^	1
3	Phenylalanine metabolism	2	9.81×10^−07^	6.38×10^−06^	0.41
4	Aminoacyl-tRNA biosynthesis	12	1.48×10^−06^	7.70×10^−06^	0.10
5	Arginine and proline metabolism	6	5.79×10^−05^	2.51×10^−04^	0.17
6	Tyrosine metabolism	1	1.32×10^−04^	4.30×10^−04^	0.14
7	Purine metabolism	3	1.95×10^−04^	5.63×10^−04^	0.11
8	Glycine, serine and threonine metabolism	6	2.27×10^−04^	5.90×10^−04^	0.26
9	Butanoate metabolism	1	7.38×10^−04^	1.75×10^−03^	0.03
10	Glycerophospholipid metabolism	1	8.11×10^−04^	1.76×10^−03^	0.03
11	Porphyrin and chlorophyll metabolism	1	3.07×10^−03^	6.15×10^−03^	0.02
12	Alanine, aspartate, and glutamate metabolism	3	5.64×10^−03^	1.05×10^−02^	0.26
13	Methane metabolism	1	1.02×10^−02^	1.47×10^−02^	0.4
14	Taurine and hypotaurine metabolism	1	2.79×10^−02^	3.63×10^−02^	0.2
15	Valine, leucine, and isoleucine biosynthesis	3	4.64×10^−02^	5.24×10^−02^	0.67

^a^ “Hits” is the number of matched compounds from the uploaded data in the pathway.

^b^ “*p*” is the original *p* value calculated from pathway enrichment analysis.

^c^ “Impact” is the pathway impact value calculated from the pathway topological analysis.

The increase of glutamic acid, γ-aminobutyrate, and histidine in all PCB95 groups, coupled with the increase of glutamine, pyroglutamic acid, tyrosine, aspartate acid, and phenylalanine in the different groups, indicated dysfunction of glutamate-related metabolism in the nervous system. Glutamic acid (Glu), a nonessential amino acid that excites the synapses in the nervous system, is the most abundant neurotransmitter [22,23]. It is involved in a variety of physiological functions, including synaptic plasticity and cognitive function [24]. In addition, Glu can be used for the synthesis of glutamine, which has roles in immune regulation and is required for lymphocyte secretion and proliferation. Glutamine also improves antioxidant capacity, thereby maintaining the stability of the cell membrane and protein structure and protecting vital organs such as the liver, lungs, and intestines [25]. Therefore, the observed increases in Glu and glutamine provide evidence for the perturbation of the nervous and immune systems. A significant increase (*p*< 0.05) in γ-aminobutyrate (GABA), similar to that of Glu, was also observed in all PCB95 groups. GABA, a metabolite of Glu, is a type of inhibitory neurotransmitter [26] that has an inhibitory effect on nerve cells overexcitement and plays an important role in suppressing the central nervous system and regulating synapse formation [27]. Therefore, the lack of GABA maybe induces anxiety, fatigue, and low resistance. Aspartate acid is a ligand for NMDA receptors, and maybe serves as a modulator in neural development process. What’s more, aspartate acid has an important role in promoting mitochondria energy metabolism and reducing the liver damage caused by alcohol [28]. Disorders of phenylalanine, tryptophan, and tyrosine biosynthesis and phenylalanine metabolism further demonstrate nervous system turbulence. Tryptophan is an essential amino acid that is metabolized via a pathway involving a variety of neural activity metabolites and is a precursor of the neurotransmitters serotonin and melatonin [29]. Tyrosine is the precursor of the neurotransmitter dopamine, which can then be converted into catecholamines. Similarly, taurine levels were also significantly increased after treatment with PCB95. Taurine is a kind of neurotransmitter involved in the growth of the nervous system, development of the retina, and absorption of lipids in the digestive tract [30].

The increased levels of serine, threonine, cysteine, tryptophan, betaine, and choline in the PCB95 groups suggest disorders of glycine, serine, threonine, and glutathione metabolism, indicating an increase in stress reactions, energy metabolism, and stimulation of nervous and immune systems. Serine is the precursor to several amino acids, including glycine, cysteine and tryptophan, and also serves as a neuromodulator by coactivating NMDA receptors and inducing the opening of these receptors upon binding. Beyond the central nervous system, D-serine signal transfer plays a signaling role in peripheral tissues and organs such as cartilage, kidney and corpus cavernosum [31–33]. Cysteine, an important sulfur-containing amino acid, serves as a precursor to the antioxidant glutathione and iron-sulfur clusters, which improve inflammation and adjust the defense mechanism of the organism. N-acetylcysteine has a protective effect against hepatic lipid accumulation in rats exposed to PCB126 [34]. Moreover, N-acetylcysteine supplementation reduces the toxicity of PCBs in human breast and prostate epithelial cells and in porcine vascular endothelial cells [35]. The underlying mechanism appears to be independent of N-acetylcysteine as a source of cysteine or a precursor of glutathione. Betaine mainly acts as a methyl donor and participates in protein, amino acid, lipid, and nucleic acid metabolism. In addition, betaine exerts an anti-stress effect by promoting the transformation of homocysteine to methionine and strengthening its central inhibitory effects [36]. Choline has an affinity for fatty acids, including promoting fatty acid transportation in the form of phospholipids or preventing abnormal accumulation of fatty acids by improving their utilization in the liver [37]. The increase of betaine and choline may indicate an increase in fatty acids, which further confirms an abnormal induction of stress reactions and energy metabolism in zebrafish embryos after treatment with PCB95.

Branched-chain amino acid (BCAA) metabolism disturbance was observed in the racemic group and the (-)-PCB95 group, as indicated by increasing valine, leucine, and isoleucine levels. Recent studies have suggested that BCAAs play a vital role in skeletal muscle and the central nervous system. Because BCAA catabolism mainly occurs in skeletal muscles, when the body experiences infection or disease, a large number of muscle proteins decompose into BCAAs as the main source of maintaining the body's energy [38]. Furthermore, BCAAs are involved in maintaining the neurotransmitter glutamate at a relatively constant level. Excess BCAAs and their derivatives result in neural dysfunction, and humans may be more susceptible to excess BCAAs than rats [39].

Excess lysine and pipecolic acid were also observed in the racemic and (-)-PCB95 groups and are probably related to anxiety, immune disorder, and liver injury. Lysine plays an important role in building muscle proteins, recovering from surgery or sports injuries and the body's production of enzymes and antibodies. Lysine exhibits anxiolytic action via its effects on serotonin receptors [40] and is related to immunity in chickens [41]. Pipecolic acid is a metabolite of lysine and abnormal increases in pipecolic acid are frequently reported in liver cirrhosis and other liver injuries [42–44].

## Conclusion

In this report, we performed a metabolomics study of zebrafish embryos after exposure to different forms of chiral PCB95 using UPLC-MS/MS combined with multivariate data analysis. This method allowed for detection of 60 metabolites, and the PLS-DA results demonstrated that (-)- and (+)-PCB95 had more prominent effects in zebrafish embryos than the racemate. A total of 26 metabolites, including amino acids, organic acids, nucleosides, betaine, and choline, were selected as potential marker metabolites, and 22 metabolites were identified and quantified by comparison with standards. The biomarker metabolites exhibited different dose-response relationships with increase of doses. Most of the enhanced metabolites tended to increase gradually from the control group to the middle-dose group and then decrease slightly in the high-dose group. Furthermore, the variations of biomarkers were related to the enantiomer-specific variation and enantiomer-specific structures, thus demonstrating enantioselective toxic effects of chiral PCB95 in zebrafish embryos. Fifteen metabolic pathways were significantly affected by PCB95, including glutathione metabolism, phenylalanine, tyrosine, and tryptophan biosynthesis, phenylalanine metabolism, and valine, leucine, and isoleucine biosynthesis. Many nervous and immune system-related metabolites were confirmed to be significantly altered by exposure to PCB95, indicating that the toxic effects of PCB95 may be related to its interactions with the nervous and immune systems, disorders of energy metabolism, and disruption of liver function.

## References

[1] Harrison PTC, Holmes P, Humfrey CD. Reproductive health in humans and wildlife: are adverse trends associated with environmental chemical exposure. Sci Total Environ 1997; 205: 97–106. 9372623 10.1016/s0048-9697(97)00212-x

[2] Kuratsune M, Yoshimura T, Matsuzaka J, Yamaguchi A. Epidemiologic study on Yusho, a poisoning caused by ingestion of rice oil contaminated with a commercial brand of polychlorinated biphenyls. Environ. Health Perspect 1972; 1: 119–128. 17539070 10.1289/ehp.7201119 PMC1474867

[3] Zhang P, Song JM, Liu ZG, Zheng GX, Zhang NX, He ZP. PCBs and its coupling with eco-environments in Southern Yellow Sea surface sediments. Mar Pollut Bull 2007; 54: 1105–1115. 17597164 10.1016/j.marpolbul.2007.05.005

[4] Wong CS, Garrison Aw, Foreman WT. Enantiomeric composition of chiral polychlorinated biphenyl atropisomers in aquatic and riparian biota. Sci Total Environ 2001; 35: 2448–2454. 10.1021/es0018872 11432547

[5] Püttmann M, Mannschreck A, Oesch F, Robertson L. Chiral effects in the induction of drug-metabolizing enzymes using synthetic atropisomers of polychlorinated biphenyls (PCBs). Biochem Pharmacol 1989; 38: 1345–1352. 2495802 10.1016/0006-2952(89)90342-0

[6] Pavagadhi S, Natera SHA, Roessner U, Balasubramanian R. Insights into lipidomic perturbations in zebrafish tissues upon exposure to microcystin-LR and microcystin-RR. Environ Sci Technol 2013; 47: 14376–14384. 10.1021/es4004125 24152164

[7] Li ZG, Qin TC, Wang KJ, Hackenberg M, Yan J, Gao Y, et al Integrated microRNA, mRNA and protein expression profiling reveals microRNA regulatory networks in rat kidney treated with a carcinogenic dose of aristolochic acid. BMC Genomics 2015; 16: 365 10.1186/s12864-015-1516-2 25952319 PMC4456708

[8] Lieschke GJ, Currie PD. Animal models of human disease:zebrafish swim into view. Nat Rev Genet 2007; 8: 353–367. 17440532 10.1038/nrg2091

[9] Best JD, Aldterton WK. Zebrafish: an in vivo model for thestudy of neurological diseases. Neuropsychiatr. Dis Treat 2004; 4: 567–576. 10.2147/ndt.s2056 PMC2526373 18830398

[10] Chen R, Wang YM, Wang JS, Lu CF, Zhang FX, Hu CX, et al Study on the toxicity of polychlorinated biphenyls and dioxin on rats by liquid chromatography-mass spectrometry based metabonomics. Environ Chem 2013; 32: 1226–1235.

[11] Xu NN, Mu PQ, Jia Q, Chai TT, Yin ZQ, Yang SM, et al Comparative enantioseparation of 19 chiral polychlorinated biphenyls with chiral stationary phase of polysaccharides derivatives. Chinese J Anal Chem 2015; 34: 795–801.

[12] Westerfield M. The zebrafish book: A guide for the laboratory use of zebrafish (Danio rerio). 2000.

[13] Busquet F, Strecker R, Rawlings JM, Belanger SE, Braunbeck T, Carr GJ, et al OECD validation study to assess intra- and inter-laboratory reproducibility of the zebrafish embryo toxicity test for acute aquatic toxicity testing. Regul Toxicol Pharm 2014; 69: 496–511. 10.1016/j.yrtph.2014.05.018 24874798

[14] Wu HF, Southam A, Hines A, Viant MR. High-throughput tissue extraction protocol for NMR-and MS-based metabolomics. Anal Biochem 2008; 372: 204–212. 17963684 10.1016/j.ab.2007.10.002

[15] Bligh EC, Dyer WJ. A rapid method of total lipid extraction and purification. Can J Biochem Phys 1959; 37: 911–917. 10.1139/o59-099 13671378

[16] Kim HJ, Seo YT, Park S, Jeong SH, Kim MK, Jang YP. DART—TOF—MS based metabolomics study for the discriminationanalysis of geographical origin of Angelica gigas roots collectedfrom Korea and China. Metabolomics 2015; 11: 64–70.

[17] Sun L, Li J, Zhou KJ, Zhang M, Yang JL, Li Y, et al Metabolomic analysis reveals metabolic disturbance inthe cortex and hippocampus of subchronic MK-801 treated rats. PLoS One 2013; 8: e60598 10.1371/journal.pone.0060598 23577129 PMC3618452

[18] Yang J, Zhao XJ, Liu XL, Wang C, Gao P, Wang JS. High performance liquid chromatography mass spectrometry for metabonomics: potential biomarkers for acute deterioration of liver function in chronic hepatitis B. J Proteome Res 2006; 5: 554–561. 16512670 10.1021/pr050364w

[19] Umetrics. User’s Guide to SIMCA-P: Umetrics Inc 2008.

[20] Reich S, Jiminez B, Marsili L, et al Congener specific determination and enantiomeric ratios of chiral polychlorinated biphenyls in striped dolphins (Stenella coeruleoalba) from the Mediterranean Sea. Environ Sci Technol 1999; 33: 1787–1793.

[21] Izabela KK, Michael WD, Lehmler HJ. Gas Chromatographic Analysis with Chiral Cyclodextrin Phases Reveals the Enantioselective Formation of Hydroxylated Polychlorinated Biphenyls by Rat Liver Microsomes. Environ Sci Technol 2011; 45: 9590–9596. 10.1021/es2014727 21966948 PMC3216237

[22] Niciu MJ, Kelmendi B, Sanacora G. Overview of glutamatergic neurotransmission in the nervous system. Pharmacol Biochem Behav 2012; 100: 656–664. 10.1016/j.pbb.2011.08.008 21889952 PMC3253893

[23] meldrum BS. Glutamate as a neurotransmitter in the brain: Review of physiology and pathology. Nut 2000; 130: 1007S–1015S. 10.1093/jn/130.4.1007S 10736372

[24] McEntee WJ, Crook TH. Glutamate-Its role in learning, memory and the aging brain. Psychopharmacology 1993; 111: 391–401. 7870979 10.1007/BF02253527

[25] Wang B, Wu GY, Zhou ZG, Dai ZL, Sun YL, Ji Y, et al Glutamine and intestinal barrier function. Amino Acids 2015; 47: 2143–2154. 10.1007/s00726-014-1773-4 24965526

[26] Owens DF, Kriegstein AR. Is there more to GABA than synaptic inhibition.Nat Rev Neurosci 2002; 3: 715–727. 12209120 10.1038/nrn919

[27] Yehezkel BA. Excitatory actions of GABA during development: The nature of the nurture. Nat Rev Neurosci 2002; 3: 728–739. 12209121 10.1038/nrn920

[28] Kim PM, Duan X, Huang AS, Liu CY, Ming GL, Song HJ, et al Aspartate racemase, generating neuronal D-aspartate, regulates adult neurogenesis. PNAS 2009; 107: 3175–3179. 10.1073/pnas.0914706107 PMC2840285 20133766

[29] Genilza SM, Adriana PC, Everton WRS, Oliveira, Adriano BS. Tryptophan: A proposal of the mechanism of thermal decomposition. J Therm Anal Calorim 2015; 122:1395–1401.

[30] Madura JD, Lombardini JB, Brigg JM. Physical and structural properties of taurine and taurine analogues. Amino Acids 1997; 13: 131–139.

[31] Takarada T, Hinoi E, Takahata Y, Yoneda Y. Serine racemase suppresses chondrogenic differentiation in cartilage in a Sox9-dependent manner. J Cell Physiol 2008; 215 (2): 320–328. 17929246 10.1002/jcp.21310

[32] Ma MC, Huang HS, Chen YS, Lee SH. Mechanosensitive N-methyl-D-aspartate receptors contribute to sensory activation in the rat renal pelvis. Hypertension 2008; 52 (5): 938–944. 10.1161/HYPERTENSIONAHA.108.114116 18809793

[33] Ghasemi M, Rezania F, Lewin J, Moore KP, Mani AR. D-Serine modulates neurogenic relaxation in rat corpus cavernosum. Biochem Pharmacol 2010; 79 (12): 1791–1796. 10.1016/j.bcp.2010.02.007 20170643

[34] Lai IK, Dhakala K, Gadupudia GS, Lia M, Ludewiga G, Robertson LW, et al N-acetylcysteine (NAC) diminishes the severity of PCB 126-induced fatty liver inmale rodents. Toxicology 2012; 302: 25–33. 10.1016/j.tox.2012.07.007 22824115 PMC3438370

[35] Slim R, Toborek M, Robertson LW, Lehmler HJ, Hennig B. Cellular glutathionestatus modulates polychlorinated biphenyl-induced stress response and apoptosis in vascular endothelial cells. Toxicol Appl Pharmacol 2000; 166: 36–42. 10873716 10.1006/taap.2000.8944

[36] Balaraman G, Rangasamy A, Thandayan LP. Studies on the protective effects of betaine against oxidative damage during experimentally induced restraint stress in Wistar albino rats. Cell Stress Chaperon 2011; 16: 641–652. 10.1007/s12192-011-0276-4 PMC3220389 21717086

[37] Ueland PM. Choline and betaine in health and disease. J Inherit Metab Dis 2011; 34: 3–15. 10.1007/s10545-010-9088-4 20446114

[38] Hatazawa Y, Tadaishi M, Nagaike Y, Morita A, Ogawa Y, Ezaki O. PGC-1α-Mediated Branched-Chain Amino Acid Metabolism in the Skeletal Muscle. PLoS One 2014; 9(3): e91006 10.1371/journal.pone.0091006 24638054 PMC3956461

[39] Susan MH, Andrew JS, Kathryn FL. Branched-Chain Amino Acid Metabolism: Implications for EstablishingSafe Intakes. J Nutr 2005; 135: 1557s–1564s. 15930469 10.1093/jn/135.6.1557S

[40] Smriga Kameishi, Uneyama Torii. Dietary L-Lysine Deficiency Increases Stress-Induced Anxiety and Fecal Excretion in Rats. J Nutr 2002; 132 (12): 3744–3746. 12468617 10.1093/jn/132.12.3744

[41] Chen C, Sander JE, Dale NM. The effect of dietary lysine deficiency on the immune response to Newcastle disease vaccination in chickens. Avian Dis 2003; 47 (4): 1346–1351. 14708981 10.1637/7008

[42] Fujita T, Hada T, Higashino K. Origin of D- and L-pipecolic acid in human physiological fluids: a study of the catabolic mechanism to pipecolic acid using the lysine loading test. Clin Chim Acta 1999; 287: 145–156. 10509903 10.1016/s0009-8981(99)00129-1

[43] Matsuda Y, Fujita T, Hada T, Higashino K. Comparative study on the correlation of plasma gamma-aminobutyric acid and pipecolic acid with liver function in patients with liver cirrhosis. Hepatol Res 2000; 18(2): 132–140. 10936564 10.1016/s1386-6346(99)00097-2

[44] Yao WF, Gu HW, Zhu JJ, Barding G, Cheng HB, Bao BH, et al Integrated plasma and urine metabolomics coupledwith HPLC/QTOF-MS and chemometric analysis on potential biomarkers in liver injury and hepatoprotective effectsof Er-Zhi-Wan. Anal Bioanal Chem 2014; 406: 7367–737. 10.1007/s00216-014-8169-x 25245419

